# Local mRNA Delivery from Nanocomposites Made of Gelatin and Hydroxyapatite Nanoparticles

**DOI:** 10.1021/acsami.4c12721

**Published:** 2024-09-16

**Authors:** Lea Andrée, Rik Oude Egberink, Renée Heesakkers, Ceri-Anne E. Suurmond, Lucas S. Joziasse, Masoomeh Khalifeh, Rong Wang, Fang Yang, Roland Brock, Sander C. G. Leeuwenburgh

**Affiliations:** † Department of Dentistry—Regenerative Biomaterials, Radboud University Medical Center, Philips van Leydenlaan 25, 6525 EX Nijmegen, The Netherlands; ‡ Department of Medical BioSciences, Radboud University Medical Center, Geert Grooteplein 28, 6525 GA Nijmegen, The Netherlands; § Department of Medical Biochemistry, College of Medicine and Medical Sciences, Arabian Gulf University, Manama 329, Bahrain

**Keywords:** gelatin nanoparticles, hydroxyapatite nanoparticles, nanocomposite, mRNA delivery, lipid-coated calcium phosphate nanoparticles, transfection

## Abstract

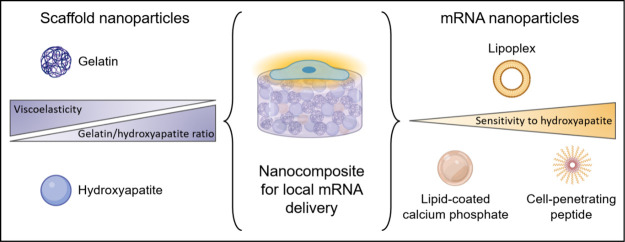

Local delivery of messenger ribonucleic acid (mRNA) is increasingly being advocated as a promising new strategy to enhance the performance of biomaterials. While extensive research has been dedicated to the complexation of these oligonucleotides into nanoparticles to facilitate systemic delivery, research on developing suitable biomaterial carriers for the local delivery of mRNA is still scarce. So far, mRNA-nanoparticles (mRNA-NPs) are mainly loaded into traditional polymeric hydrogels. Here, we show that calcium phosphate nanoparticles can be used for both reinforcement of nanoparticle-based hydrogels and the complexation of mRNA. mRNA was incorporated into lipid-coated calcium phosphate nanoparticles (LCPs) formulated with a fusogenic ionizable lipid in the outer layer of the lipid coat. Nanocomposites of gelatin and hydroxyapatite nanoparticles were prepared at various ratios. Higher hydroxyapatite nanoparticle content increased the viscoelastic properties of the nanocomposite but did not affect its self-healing ability. Combination of these nanocomposites with peptide, lipid, and the LCP mRNA formulations achieved local mRNA release as demonstrated by protein expression in cells in contact with the biomaterials. The LCP-based formulation was superior to the other formulations by showing less sensitivity to hydroxyapatite and the highest cytocompatibility.

## Introduction

Large bone defects, resulting from, e.g., trauma, tumor resection, or congenital malformations, pose a significant challenge in various surgical disciplines. Biomaterials, often combined with proteinaceous growth factors (GFs), are routinely used in clinics. However, the therapeutic efficacy of local GF delivery from biomaterial carriers is limited by their rapid clearance from the defect site, necessitating their administration in supraphysiological amounts.^1,2^ For bone morphogenetic protein 2 (BMP-2), the most used GF in bone regeneration, severe (systemic) side effects, including osteolysis, dysphagia, and damage of nerve tissue, were observed in the clinic when applied in high concentrations.^3,4^ This approach increases treatment costs and raises serious concerns regarding the clinical safety of GF delivery. Messenger ribonucleic acid (mRNA) is increasingly considered as a promising alternative to GFs with a recent *in vivo* study in rats showing that mRNA coding for BMP-2 outperformed proteinaceous BMP-2 and led to bone regeneration without the formation of a massive callus observed for proteinaceous BMP-2.^5^

In contrast to delivery of GFs, mRNA therapy leverages the cell’s own translation machinery to stimulate the production of endogenous proteins.^6^ However, achieving successful transfection requires maintaining mRNA stability and ensuring a high transfection efficiency. To this end, a plethora of transfection agents have been developed based on lipids, peptides, and polymers.^7,8^ While extensive research has been dedicated to the development of mRNA formulations to facilitate systemic delivery, research on developing suitable biomaterial carriers for local delivery of mRNA is still scarce. mRNA-nanoparticles (mRNA-NPs) are formed through charge-driven noncovalent interactions of the negatively charged oligonucleotides with the positively charged carrier material. The requirement for low-temperature storage underlines the sensitivity of classical lipid-based formulations to degradation.^9^ For sustained, local delivery, the mRNA-NPs must be compatible with the biomaterials and withstand premature decomplexation. So far, mRNA-NPs have only been combined with traditional polymeric hydrogels, reviewed elsewhere.^6^ Unfortunately, these hydrogels are mechanically weak due to their high water content and offer poor spatiotemporal control over mRNA release characteristics due to their large mesh size which often leads to undesired burst-type release profiles.^10^

Alternatively, particulate hydrogels or colloidal hydrogels represent a versatile solution, offering more freedom in biomaterial design. Particulate hydrogels are fully assembled from nanoparticles that interact with each other to form a network structure.^11^ This bottom-up approach allows for the customization of biomaterials with unique properties by combining different types of nanoparticles. For example, the incorporation of inorganic nanoparticles (silica, bioglass, calcium phosphate) can be used to mechanically strengthen hydrogels and match their mechanical properties more closely to the target tissue.^11−13^ The freedom of design and easy incorporation of different nanoparticle types render particulate hydrogels as promising carrier materials for mRNA-NPs.

Here, we designed a particulate nanocomposite based on gelatin (GNP) and hydroxyapatite nanoparticles (nHA) to enable local mRNA delivery. These two types of nanoparticles were selected as building blocks given their similarity to the composition of the extracellular bone matrix as well as their potential to deliver mRNA. Gelatin, derived from collagen—the primary organic phase of bone^14^—offers inherent biocompatibility and biodegradability and provides abundant cell attachment motifs.^15^ Moreover, GNP-based hydrogels have been successfully used to achieve sustained release of therapeutic agents such as growth factors and antibiotics^16,17^ and exhibit viscoelastic properties favorable for cells.^18^ Calcium phosphates, on the other hand, are widely used in orthopedics and dentistry as bone substitutes due to their compositional similarity to bone mineral, osteoconductive capacity, i.e., their ability to promote bone formation. In the form of nanoparticles, calcium phosphates are readily internalized by cells and dissolve quickly in the acidic environment of the lysosome. Upon dissolution calcium phosphates release calcium and phosphate ions that can serve as a source for the deposition of new bone minerals.^19^ Calcium phosphate precipitation was one of the first methods to afford cellular transfection with plasmid DNA. Inside endosomes, oligonucleotides are released due to the pH-dependent solubility of calcium phosphate. Such calcium phosphate precipitations have also been tested for antisense oligodeoxynucleotides, siRNA, miRNA, and mRNA.^20−25^ However, bare calcium phosphate nanoparticles are colloidally instable, leading to agglomeration and poor control over pharmacokinetic profiles.^26^ Lately, this concept has been revisited in the form of lipid-coated calcium phosphate nanoparticles (LCPs) in which a calcium phosphate core is encapsulated by an asymmetric lipid bilayer which provides colloidal stability and enhances cellular uptake.^23,26^ Although the lipid composition is similar to the clinically approved lipid nanoparticle-based mRNA vaccines, the ionizable lipids in the LCPs are not involved in the electrostatic complexation of mRNA. Instead, the mRNA-containing calcium phosphate core of the LCP is decorated with an asymmetrical lipid bilayer, consisting of a negatively charged inner layer of DOPA and an outer layer of DSPE-PEG200 and DOTAP that additionally contains an ionizable lipid component. To date, only three ionizable cationic lipids have been clinically approved for RNA delivery: DLinDMA,^27^ ALC03-15,^28^ and SM-105.^29^ Additional functionalities such as poly(ethylene glycol) (PEG) or antibodies can easily be added to the lipid bilayer to improve circulation time *in vivo* and targeted delivery of these LCPs, respectively.^30^ Moreover, by using ionizable lipids in the outer lipid layer, cellular uptake and endosomal release can be enhanced to improve mRNA delivery.^31^

In this study, we aimed to obtain proof-of-principle for the development of nanocomposites made of GNPs and nHA to facilitate local delivery of mRNA. To this end, GNPs and nHA were synthesized, characterized regarding their physicochemical characteristics, and subsequently assembled into composites with different GNP-to-nHA weight ratios to optimize their viscoelastic properties and cytocompatibility. The nanocomposites were tested for their ability to deliver mRNA complexed with either peptides, lipids, or LCPs as transfection agents. The LCP-formulated material provided uniformly high transfection efficiency independent of the hydroxyapatite content.

## Methods

### Synthesis of mRNA-Nanoparticles

The cell-penetrating peptide PepFect14 (PF14) was purchased from EMC microcollections (Tübingen, Germany). The peptide has the following sequence: Stearyl-AlaGlyTyrLeuLeuGlyLysLeuLeuOrnOrnLeuAlaAlaAlaAlaLeuOrnOrnLeuLeu-NH2, where “Orn” denotes the non-proteinogenic amino acid ornithine and “-NH2” indicates a C-terminal amidation. PF14 was dissolved in Milli-Q (MQ) water, stored in Protein LoBind tubes (Eppendorf), and incubated at room temperature (RT) for 20 min under gentle agitation before aliquots were snap-frozen in liquid nitrogen and stored at −20 °C. mRNA coding for secreted nanoluciferase (SecNLuc) was purchased from RIBOPRO (Oss, The Netherlands). All mRNA was aliquoted at 100 ng/μL in nuclease-free water (Thermo Fisher Scientific) in DNA LoBind tubes (Eppendorf), snap-frozen in liquid nitrogen, and stored at −80 °C until use. Before use, the mRNA solutions were thawed and kept on ice. PF14 nanoparticles were prepared as described previously.^32−34^ For the formation of cationic lipid-based complexes (lipoplexes), Lipofectamine MessengerMAX (LMM; Thermo Fisher Scientific) was used according to the manufacturer’s instructions. In short, LMM was incubated in Opti-MEM (Gibco, cat. no. 11058021) for 10 min at room temperature (RT). The appropriate amount of mRNA solution was diluted in Opti-MEM and incubated with LMM for at least 5 min at RT.

### Synthesis of mRNA Lipid-Coated Calcium Phosphate Nanoparticles

mRNA lipid-coated calcium phosphate nanoparticles (LCPs) were prepared as described previously^31^ with minor adaptations. SM-102 was chosen as the ionizable lipid in the outer layer as this lipid has been shown to outperform the other two clinically approved ionizable lipids, DLinDMA and ALC03-15.^35,36^ First, 60 μL of 2.5 M calcium chloride (Sigma-Aldrich) was mixed with 50 μL of 100 ng/μL mRNA under gentle stirring (∼3 min at 250 rpm) and subsequently added to 2 mL of cyclohexane:igepal CO-520 (70:30 v/v%, both from Sigma-Aldrich). Meanwhile, 110 μL of 12.5 mM of disodium phosphate (pH 9.0, Sigma-Aldrich) was dispersed into 2 mL of the cyclohexane:igepal mixture. After gentle stirring for 15 min at RT, 2 mL of the phosphate phase was added dropwise to the calcium-containing vial while stirring. After brief mixing, 40 μL of 20 mM dioleoyl phosphatidic acid (DOPA; Avanti, Cat. No. 840875P) was added in a dropwise manner and subsequently stirred for 20 min, while taking special care to avoid any foam formation. The resulting microemulsion was broken with the addition of 4 mL of absolute EtOH (ThermoFisher Scientific), mixed at full speed (∼1500 rpm) for 5 min, subsequently centrifuged for 20 min at 10,000 rcf, and finally washed with absolute EtOH for a total of 3 washes. Then, residual EtOH was removed by gently flushing a stream of argon gas over the pellet. Thereafter, the pellet was redissolved in chloroform (Sigma-Aldrich). For the outer layer lipids, 14 μL of 20 mM 1,2-dioleoyl-3-trimethylammonium–propane (DOTAP; Avanti, Cat. No. 890890P), 14 μL of 20 mM cholesterol (Sigma-Aldrich, Cat. No. C75209), and 12 μL of 1,2-distearoyl-*sn*-glycero-3-phosphoethanolamine-*N*-[amino(poly(ethylene glycol))-2000] (DSPE-PEG2000; Avanti, Cat. No. 880120P) were added per 50 μL of CaP core solution in Protein LoBind tubes (Eppendorf). Lastly, SM-102 (Cayman Chemical, Cat. No. Cay33474-) was incorporated in the outer leaflet of the LCPs to induce particle formation. Chloroform was then removed by gentle flushing over a stream of argon gas. Lastly, LCPs were rehydrated in ∼100 μL of prewarmed Milli-Q at 50 °C and briefly sonicated until a well-dispersed solution was obtained. LCPs were then stored at 4 °C until further use.

### Synthesis of Gelatin Nanoparticles

Gelatin type A (Bloom number 285, kindly provided by Rousselot BV Ghent, Belgium) was chosen for the synthesis of gelatin nanoparticles (GNPs) to obtain positively charged GNPs for interaction with negatively charged citrate-modified hydroxyapatite nanoparticles. GNPs were obtained by desolvation with ethanol. 5 g of gelatin was dissolved in 100 mL of demineralized water under stirring (500 rpm) at 40 °C. The pH was adjusted to 3.5 by adding 1.86 mL of 1 M HCl (37% fuming, Merck) whereafter 117 mg of sodium chloride (Merck) was added. This solution was stirred for 10 min, after which the stirring speed was increased to 1000 rpm and 320 mL of ethanol (99.5% Boom) was added dropwise at 5 mL/min using a syringe pump to induce nanoparticle formation through desolvation. This suspension was left to cool to room temperature for 20 min. Subsequently, the nanoparticles were cross-linked using EDC-NHS. In brief, 400 mg of EDC (Sigma-Aldrich) and 60 mg of NHS (Merck) were dissolved in 5 mL of demineralized water and added dropwise (1 drop/10 s) to the nanoparticle solution. The suspension was left to stir overnight at room temperature before purification of the nanoparticles by crossflow filtration (Hydrosart, cutoff 300 kDa, Sartorius) under the addition of 1.5 L of demineralized water. GNPs were stored in demineralized water at 4 °C until further use.

### Synthesis of Hydroxyapatite Nanoparticles

Hydroxyapatite nanoparticles (nHA) were synthesized by one-pot wet-chemical synthesis at 40 °C. An aqueous solution of 50 mM sodium phosphate (Sigma-Aldrich) was heated to 40 °C under stirring. When the temperature of 40 °C was reached, an equal amount of 83.5 mM calcium acetate (Sigma-Aldrich) was added while stirring vigorously at 1000 rpm. The solution was covered and left to stir at 500 rpm at 40 °C for 2 h. Afterward, 940 mg of tribasic sodium citrate (Merck) was added, and the reaction was left to stir for another 3 h. The nanoparticles were then collected by centrifugation (16,800 rcf, 5 min) and subsequently washed twice with demineralized water by resuspension through sonication (5 min) and centrifugation (16,800 rcf, 10 min). nHA was resuspended in demineralized water for storage at 4 °C.

### Characterization of Nanoparticles

The hydrodynamic diameters of GNPs and nHA were measured in demineralized water by dynamic light scattering (DLS) using a Malvern Zetasizer Lab (Malvern Instruments), while the zeta potential of NPs was measured in 5 mM HEPES buffer (Sigma-Aldrich) at pH 7.4. To visualize their morphology, GNPs were lyophilized in an ethanol/water mixture (30/70 v/v%), while nHA was diluted 1000× in demineralized water, and 10 μL was left to air-dry on a copper grid. Both samples were sputter-coated with gold–palladium and imaged using a scanning electron microscope (SEM; Sigma 300 field-emission scanning electron microscope, Zeiss). The average size in the dry state was determined by measuring the diameter of 100 nanoparticles in SEM images using open-source Fiji software. GNP and nHA concentrations were determined by freeze-drying two samples of 0.5 mL of NP suspension and weighing the dried powder. For nHA the dried powder was further used to analyze the molecular and crystal structure using Fourier-transform infrared (FTIR, PerkinElmer) spectroscopy and X-ray diffraction (XRD, Panalytical), respectively. mRNA-NP size and surface charge were determined in Milli-Q water by means of DLS using a NANO-flex apparatus (Microtrac MRB).

### Preparation of Nanocomposite

Nanocomposites with different GNP-to-nHA ratios were prepared for rheological analysis and cell culture studies in Minimal Essential Medium α (Gibco, MEM-α without ascorbic acid) without the addition of serum. First, GNP and nHA suspensions were mixed to reach the desired GNP/nHA weight ratios of 1:1, 2:1, and 4:1. Subsequently, the mixtures were flash-frozen with liquid nitrogen and lyophilized. GNP-nHA nanocomposites were prepared by mixing 18 wt % dried NP-mix in MEM-α in a 2 mL Eppendorf tube using centrifugation (300 rcf, 5 min). The nanocomposites were left to swell completely overnight at 4 °C.

### Rheology

The viscoelastic properties of GNP-nHA nanocomposites were determined with a TA AR2000ex rheometer (TA Instruments) using a 20 mm plate–plate geometry at a 400 μm gap width following a previously described protocol.^18^ After the application of the nanocomposites, the geometry was sealed with silicon oil to prevent water evaporation. Frequency sweeps were performed at a constant strain of 0.5% by increasing the oscillatory frequency from 0.1 to 100 rad/s. Strain sweeps were performed at a constant frequency of 1 rad/s in a range between 0.1 and 1000%. The self-healing properties of GNP-nHA nanocomposites were determined by consecutive strain sweeps up to 1000% strain, followed by recovery at 0.5% strain. The recovery percentage was calculated based on the last five values before the start of the strain sweep. All experiments were performed in triplicate at 37 °C.

### Cell Culture

The murine preosteoblast cell line MC3T3-E1 subclone 4 (CRL-2593, American Type Culture Collection) was maintained in complete medium consisting of MEM-α, supplemented with 10% FBS (Gibco) and 100 units/mL penicillin and 0.1 mg/mL streptomycin (Sigma-Aldrich). Human bone marrow-derived mesenchymal stromal cells (hBMSCs) were isolated postsurgery from iliac bone fragments of healthy donors (Department of Maxillofacial Surgery, Radboudumc, The Netherlands) after ethical approval (Commissie Mensgebonden Onderzoek: dossier number #2017-3252) as described previously.^37^ In line with the criteria as set by the International Society for Cellular Therapy (ISCT), hBMSCs were characterized immunophenotypically for the expression of characteristic MSC markers (>95% immunopositive for CD73, CD90, and CD105, and immunonegative for CD45) and the capacity to undergo osteogenic differentiation.^38^ hBMSCs were maintained in Minimal Essential Medium α (MEM-α without ascorbic acid), supplemented with 10% FBS and 100 units/mL penicillin and 0.1 mg/mL streptomycin and used from passage 2-7. These cell types were selected based on their potential for osteogenic differentiation.

### Preparation of PDMS Rings for Cell Culture

To keep the nanocomposites stable in cell culture and facilitate handling of the nanocomposites, rings with an outer diameter of 8 mm and an inner diameter of 4 mm were punched out of a 2 mm high polydimethylsiloxane (PDMS, Slygard 184 Silicone Elastomer Kit, Dow Corning) layer prepared according to the manufacturer’s protocol. In brief, ten parts of PDMS was mixed with one part curing agent by vigorous stirring and poured into a 60 mm Petri dish (Greiner). Air bubbles were removed under vacuum, and the PDMS was left to cross-link at 37 °C overnight. Rings were sterilized by being autoclaved before use.

### Cytocompatibility of Nanocomposites

The cytocompatibility of nanocomposites with different GNP-to-nHA ratios was tested by culturing MC3T3s on the surface of these nanocomposites. After preparation of the nanocomposites as described above, the nanocomposites were smeared into the PDMS rings using a sterile spatula and transferred into a 48-well plate (Greiner). 12,500 MC3T3s were seeded per gel (= 100,000 cells/cm^2^) in 10 μL complete medium and left to adhere for 4 h before gently adding 400 μL medium to the well. Nanocomposites without cells were used as a negative control for the metabolic activity and DNA assay.

#### Metabolic Activity

The metabolic activity of cells seeded on the nanocomposites was measured over time using the Alamar Blue assay after 3, 7, 14, 21, and 28 days. At the respective time points, nanocomposites were transferred into a fresh 48-well plate and a 10% (v/v) Alamar Blue suspension in complete medium was added. After 6 h of incubation, 100 μL of supernatant was transferred into a black bottom 96-well plate (Greiner), and fluorescence was read at an excitation wavelength of 560 nm and an emission wavelength of 620 nm using a spectrophotometer (Synergy HTX multimode reader, Biotek). The average signal of cell-free nanocomposites was used as a blank. The remaining supernatant was aspirated, and nanocomposites were washed thrice with Dulbecco’s phosphate buffered saline (DPBS without calcium and magnesium, Gibco) for 3 min before adding fresh medium for further culture.

#### DNA Assay

DNA content was measured after 3, 7, 14, 21, and 28 days of culture using the QuantiFluor dsDNA kit (Promega) and after dissociating the nanocomposites by lyophilization. In brief, nanocomposites were washed twice with DPBS and frozen at −20 °C. The frozen nanocomposites were removed from the PDMS rings, transferred into Eppendorf tubes, frozen at −80 °C, and lyophilized. The freeze-dried nanocomposites were mechanically pulverized into a powder, and 400 μL of Tris buffer (50 mM Tris-HCl (Merck) and 3 mM calcium chloride (Sigma-Aldrich), pH 7.8) was added per tube. Samples were freeze–thawed twice before the DNA assay. To this end, 50 μL of sample was mixed with 50 μL of QuantFluor dye solution (1:200 in Tris-EDTA buffer) and left to react for 5 min before reading the fluorescence at an excitation wavelength of 504 nm and an emission wavelength of 531 nm. Absolute DNA concentrations were calculated using a standard curve prepared with lambda standard DNA according to the manufacturer’s instructions.

### Cytocompatibility of mRNA-Nanoparticles in the Presence of Hydroxyapatite Nanoparticles

The effect of different mRNA-NPs complexed with either peptide (PF14), lipids (LMM), or lipid-coated calcium phosphate NP (LCPs) on cell viability in the presence of nHA was assessed by measuring the metabolic activity of cells 26 h post-transfection. In short, 10,000 MC3T3s or hBMSCs were seeded in 96-well plates and left to adhere overnight. The next day, 100 ng/well mRNA was added formulated as nanoparticles (PF14, LMM and LCPs), whereas nHA was added to the medium at concentrations of 0, 50, or 150 μg/mL. After 26 h, metabolic activity was measured using a resazurin-based assay as described previously.^39^ Resazurin sodium salt (Sigma-Aldrich) was dissolved in PBS and diluted 100 times to a final concentration of 100 μg/mL in a complete medium. After 2 h of incubation with cells at 37 °C, fluorescence was measured using the VICTOR X3Multilabel plate reader (PerkinElmer). After the plate was briefly shaken, resazurin was excited at 485 nm, and emission was collected from 570 to 620 nm. All samples were blanked by the average signal of the cell-free wells. Blanked data were normalized to the untreated conditions without nHA.

### Transfection in the Presence of Hydroxyapatite Nanoparticles

The effect of the presence of nHA on the transfection efficiency of mRNA-NPs was assessed using secreted luciferase (SecNLuc) mRNA. 10,000 MC3T3 cells or hBMSCs were seeded in 96-well plates 24 h pretransfection. Cells were transfected with 100 ng of SecNLuc mRNA and formulated with different complexation agents (PF14, LMM, and LCPs) at 10 ng of mRNA/μL, in complete medium supplemented with 0 mg/mL, 50 μg/mL, or 150 μg/mL nHA for 4.5 h. Luciferase expression was assessed 24 h post-transfection using the Nano-Glo Luciferase Assay (Promega, Madison, WI, Cat. No. N1130) according to the manufacturer’s instructions. Luminescence was measured after briefly shaking the plate using a VICTOR X3Multilabel plate reader (PerkinElmer).

### Transfection of Cells in Contact with Nanocomposites

For mRNA transfections in the presence of nanocomposites, the nanocomposites were formed as described above using SecNLuc-mRNA-NP formulations in Milli-Q instead of α-MEM. In brief, mRNA-containing nanocomposites were prepared by mixing an 18 wt % dried GNP-nHA-mix with SecNLuc-mRNA-NP formulations (diluted to 10 ng of mRNA/μL in Milli-Q) in a 2 mL Eppendorf tube using centrifugation (300 rcf, 5 min). For the untreated and free mRNA conditions, an equal amount of Milli-Q was added as for the other experimental conditions to create a nanocomposite with a solid content of 18 wt %. The nanocomposites were left to swell for 18 h at 4 °C. The next day, the samples were allowed to equilibrate to room temperature. The swollen nanocomposites were then aseptically smeared into PDMS rings with sterilized spatulas and transferred to 48-well plates. Then, 100,000 MC3T3 cells were added to the nanocomposites and allowed to adhere for 4 h as described above, after which an additional 300 μL of complete medium was added. Luciferase expression was assessed 24 h later.

### Statistical Analysis

Statistical analysis was carried out using Prism version 8.4 (GraphPad). Cellular metabolic activity, DNA content, and transfection data in the presence of nHA were analyzed by two-way analysis of variance (ANOVA) with Tukey multiple comparison corrections to detect differences between the different nanocomposite formulations or nHA concentrations. Transfection data of nanocomposites were analyzed by *t* tests with Bonferroni correction for multiple testing to detect differences of mRNA-containing nanocomposites compared to the controls (untreated and free mRNA), and one-way ANOVA with Games Howell correction was used to detect differences between the different mRNA-NPs. Using a Welch test, the effect of nanocomposite nHA content on transfection efficiency was tested by comparing protein expression in 1:1 and 2:1 nanocomposite formulations per mRNA-NP group. Metabolic activity experiments on nanocomposites were performed in quintuplicates (*n* = 5), while DNA content was measured in quadruplicates (*n* = 4). However, some samples were lost during culture, resulting in *n* = 4–5 for metabolic activity and *n* = 2–4 for DNA measurements. Metabolic activity and transfection in the presence of nHA were assessed in triplicates (*n* = 3). Transfection with nanocomposites was carried out in quadruplicates (*n* = 4). All data are presented as mean ± standard deviation. Significance was set at *p* < 0.05, and *p* values are reported using *p < 0.05, **p < 0.01, ***p < 0.001, and ****p < 0.0001.

## Results and Discussion

### Characterization of Nanoparticles

**Figure 1:**
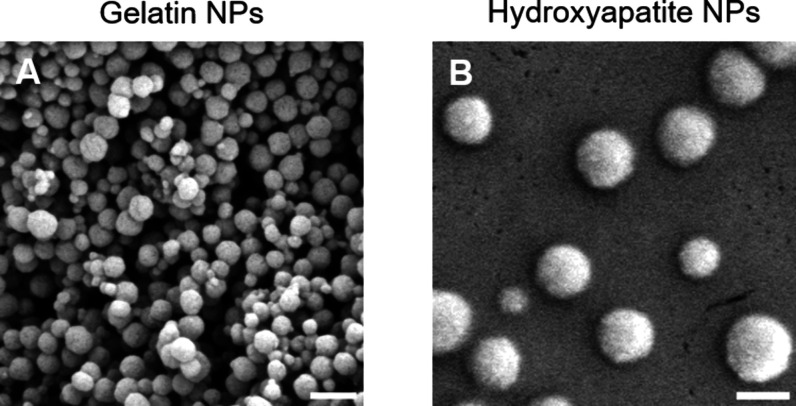
Scanning electron microscopy images of (A) lyophilized gelatin nanoparticles and (B) air-dried hydroxyapatite nanoparticles. Scale bar = 200 nm.

GNPs and nHA showed a spherical morphology with a dry size of 77 ± 15 and 123 ± 16 nm, respectively, as determined from SEM images (Figure 1 and Table 1). In the wet state, GNPs swelled substantially, reaching a hydrodynamic size of 601 ± 10 nm, while nHA remained in the range of 162 ± 3 nm as measured by DLS (Table 1 and Figure S1A). The zeta potential was +14 and −20 mV for GNPs and nHA, respectively. XRD measurements showed diffractograms corresponding to the powder diffraction pattern of hydroxyapatite (Figure S2). mRNA-NPs could not be imaged using SEM, but their hydrodynamic size and zeta potential were measured using DLS (Table 2 and Figure S1B). LCPs had a size and zeta potential of ∼93.6 ± 9.6 and +20 mV, respectively, whereas PF14 NPs were more monodisperse with a slightly smaller size of 81.7 ± 2.7 nm and a zeta potential of +26 mV. LMM particles, on the other hand, had a size of 726 ± 9.9 nm, which is about an order of magnitude larger than LCPs and PF14, as reported previously.^32,33^ The zeta potential of the LMM could not be measured.

**Table 1:** Characteristics of Gelatin and Hydroxyapatite Nanoparticles

	size_SEM_ (nm)	size_DLS_ (nm)	PDI	zeta (mV)
gelatin	77 ± 15	601 ± 10	0.723 ± 0.472	+14 ± 1
hydroxyapatite	123 ± 16	162 ± 3	0.153 ± 0.024	–20 ± 1

**Table 2:** Characteristics of Peptide, Lipid, and Lipid-Coated Calcium Phosphate (LCP) mRNA-Nanoparticles

	size_DLS_ (nm)	PDI	zeta (mV)
PepFect14	82 ± 3	0.194 ± 0.018	26.4 ± 5.3
Lipofectamine Messenger Max	570 ± 28	0.322 ± 0.042	n/a
LCP	94 ± 3	0.388 ± 0.021	20.0 ± 0.7

### Characterization of Nanocomposites

**Figure 2:**
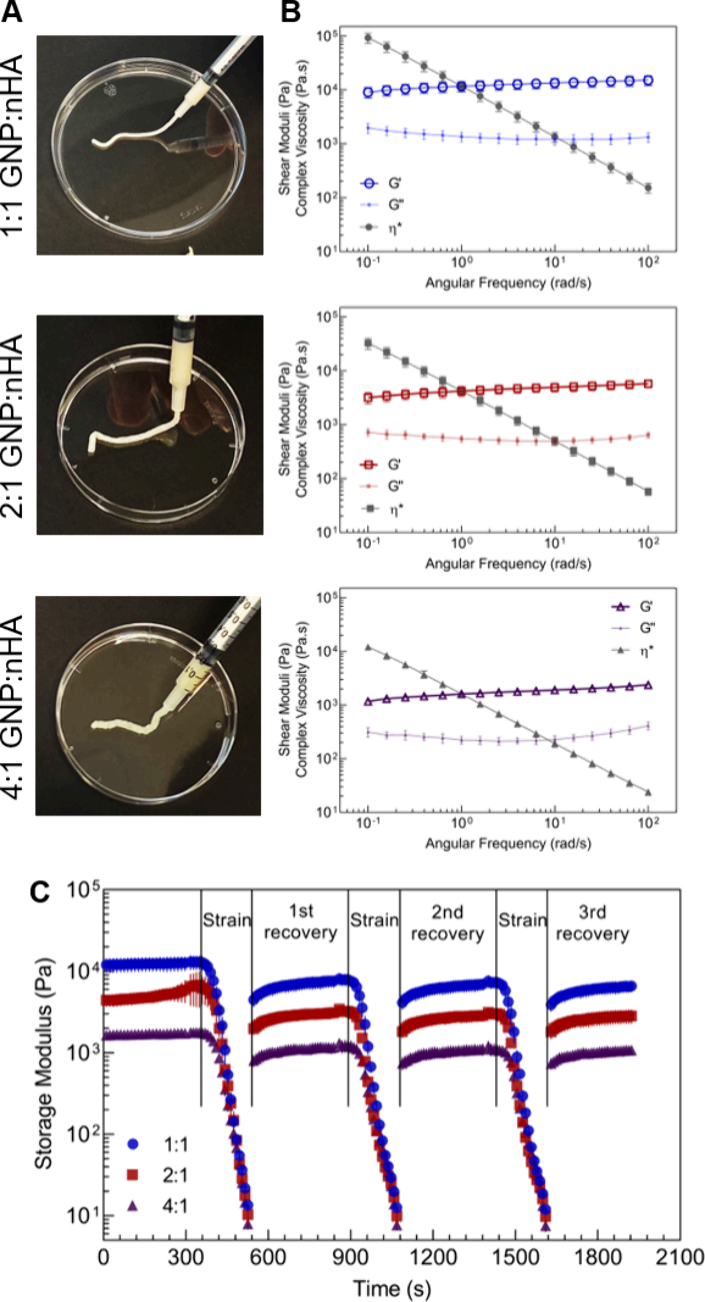
(A) Photographs, (B) frequency sweeps, and (C) time sweeps with consecutive strain–recovery cycle of 18 wt % nanocomposites at different gelatin-to-hydroxyapatite nanoparticle ratios (GNP:nHA).

When combining GNPs and nHA at 18 wt %, a paste-like water-swollen nanocomposite was formed at all three GNP-to-nHA ratios (1:1, 2:1, 4:1), and the nanocomposites could be extruded through a syringe (Figure 2A), similar to previously designed formulations solely composed of GNPs, or mixtures of GNPs and bioglass particles.^13,40^ Upon rheological characterization, the three formulations showed stable shear moduli over the measured frequency range with a storage modulus higher than the loss modulus, indicative of solid-like elastic behavior. Storage moduli increased with increasing nHA content (Figure 2B), confirming the reinforcing effect of nHA on the mechanical properties of the resulting nanocomposites, as previously reported for GNPs hydrogels reinforced with silica nanoparticles.^11^ The storage moduli of the nanocomposites were between 1 and 10 kPa while the storage modulus of bone tissue is reported to be in the range of 10 GPa.^41^ However, it is important to note that hard tissues such as bone evolve from softer tissues that progressively stiffen during development. Likewise, after fracture, bone regeneration is preceded by softer tissues (hematoma, granulation tissue, callus).^42^ Hence, the soft nature of the nanocomposite may support bone regeneration. Nevertheless, the fracture will need additional mechanical support in the form of screws and plates to stabilize the bone ends. Another aspect regarding the applicability of nanocomposites for bone regeneration is their administration at the defect site. All three formulations showed shear-thinning behavior, as evidenced by the decreasing complex viscosity with increasing angular frequency (Figure 2B), indicating their potential for minimally invasive application. Moreover, the three nanocomposites showed recovery of the storage modulus of about 57–67% after the first cycle of destructive shearing (Figure 2C and Table S1), which is a lower self-healing percentage than reported for colloidal gels solely composed of GNPs.^18^ Of note, nHA content did not significantly affect the self-healing ability. With an increasing number of shear–recovery cycles, the recovery improved up to 95–98% after the third destructive shear cycle. The improved self-healing is likely caused by the rearrangement of the particle network, as previously reported for hydrogels composed of GNPs and silica nanoparticles.^11^ Overall, these results confirm the successful preparation of nanocomposites made of GNPs and nHA and indicate their potential for minimally invasive application.

### Cytocompatibility of Nanocomposites

**Figure 3:**
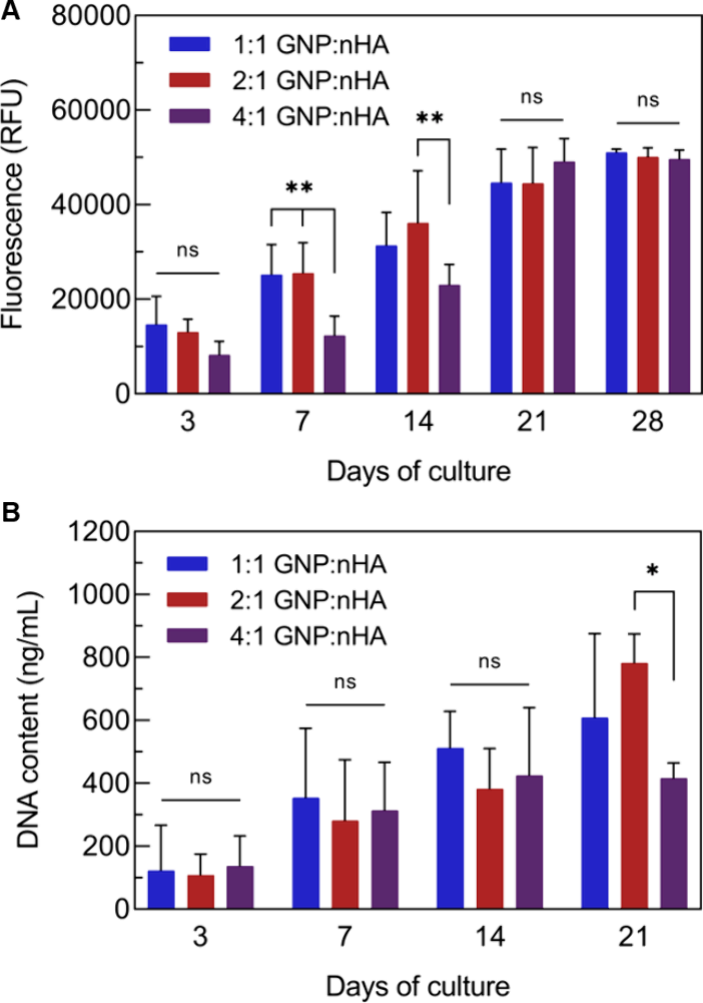
Cytocompatibility of mRNA-free nanocomposites of different gelatin-to-hydroxyapatite nanoparticle ratios (GNP:nHA) as measured by (A) metabolic activity and (B) DNA content in MC3T3 cells.

We had previously confirmed the cytocompatibility of GNPs and colloidal nHA.^43^ Therefore, we here assessed the cytocompatibility of the three GNP-nHA nanocomposites by direct culture of murine preosteoblastic cells, MC3T3, on the nanocomposites. All three nanocomposites supported cell proliferation, as indicated by an increase in metabolic activity with culture time (Figure 3A). Interestingly, cells proliferated less on the 4:1 formulation compared to the other two nanocomposites, especially during the initial 3 weeks of culture. An excessively low storage modulus might cause this slower growth behavior on the 4:1 formulation compared to the other two nanocomposites, as we have previously demonstrated that MC3T3 cells grow better on stiffer gels.^44^ While DNA content measurements showed no significant differences across the groups for most of the time points, a notable reduction was observed on day 21 for the 4:1 formulation compared to that of the other two nanocomposites (Figure 3B). Overall, these results indicate that the nanocomposites support cell proliferation. Given the slower cell growth on the 4:1 nanocomposite, we selected the 1:1 and 2:1 formulations for subsequent transfection studies.

### Cytocompatibility and Transfection Efficiency of mRNA-Nanoparticles in the Presence of Hydroxyapatite Nanoparticles

mRNA transfection agents may show cytotoxicity at high concentrations due to their ability to interact with and also disrupt membranes.^45^ Also, nHA can be cytotoxic dependent on various material parameters and concentrations.^19^ Moreover, we have previously shown that the transfection efficiency of peptide- or lipid-based complexation agents can be influenced by the presence of charged nanoparticles such as GNPs.^32^ Since the nHA particles employed herein are negatively charged, we anticipated that these nanoparticles could similarly interact with these transfection agents, potentially leading to the decomplexation of mRNA and the release of cytotoxic transfection agents. Since the nanocomposites are made of GNPs and nHA, we assessed the cytocompatibility and transfection efficiency of mRNA-NPs in the presence of nHA.

**Figure 4:**
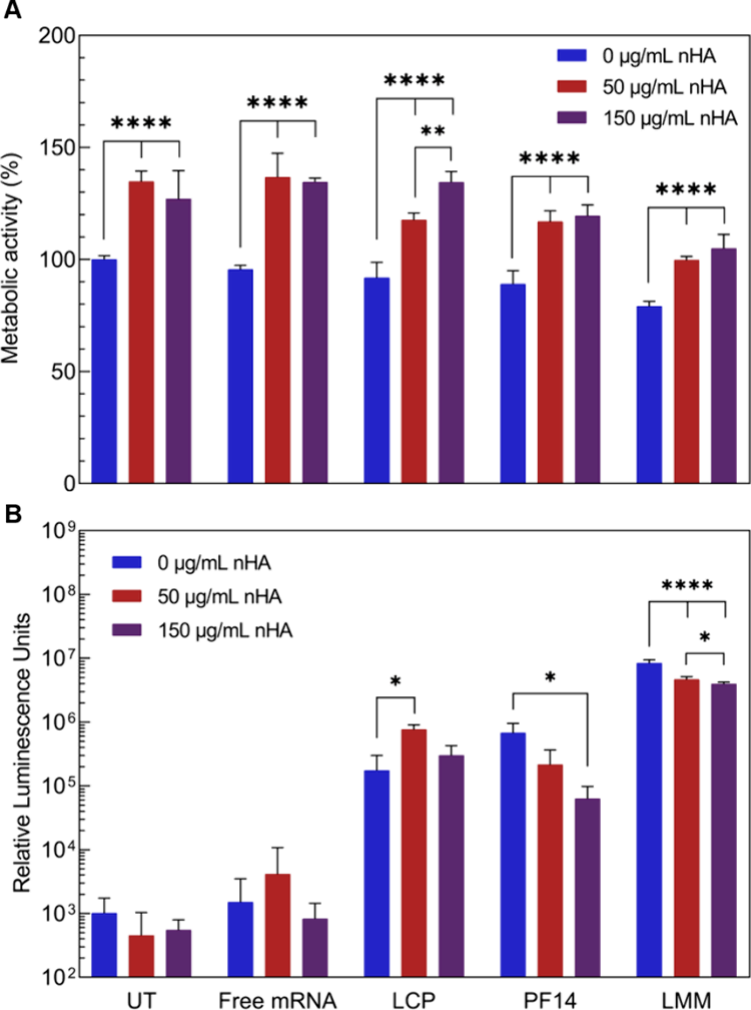
Effect of hydroxyapatite nanoparticles on (A) the cytocompatibility and (B) the transfection efficiency of mRNA-nanoparticles in MC3T3 cells. UT = untreated, LCP = lipid-coated calcium phosphate, PF14 = PepFect 14, and LMM = Lipofectamine Messenger Max.

As shown in Figure 4A, addition of nHA led to increased metabolic activity of MC3T3 cells after 24 h of exposure, independent of the presence of mRNA-NPs or the specific type of formulation (LCP, PF14, LMM). Increased metabolic activity can reflect cellular stress and thus reduced cytocompatibility.^43^ Among the tested conditions, cells exposed to PF14 or LMM tended toward lower metabolic activity than cells exposed to LCPs. However, for none of the conditions metabolic activity dropped below 77%, indicating good cell viability as also confirmed for human bone marrow-derived mesenchymal stromal cells (Figure S3A).

With regard to transfection efficiency, all mRNA formulations led to the luciferase expression above the background (Figure 4B). Notably, without nHA, PF14 and LMM showed higher expression levels than LCPs, whereas in the presence of nHA expression levels for PF14 and LMM groups decreased but remained unaffected for LCPs, which was also confirmed for hBMSCs (Figure S3B). We attribute this observation to potentially higher mRNA stability in LCPs due to the 2-fold protection by the calcium phosphate core and the lipid bilayer. Overall, these results suggest that peptide and lipid mRNA-NPs are more affected by the presence of inorganic biomaterials, as seen by the decrease in transfection efficiency with increasing nHA concentration.

### Transfection with Nanocomposites

**Figure 5:**
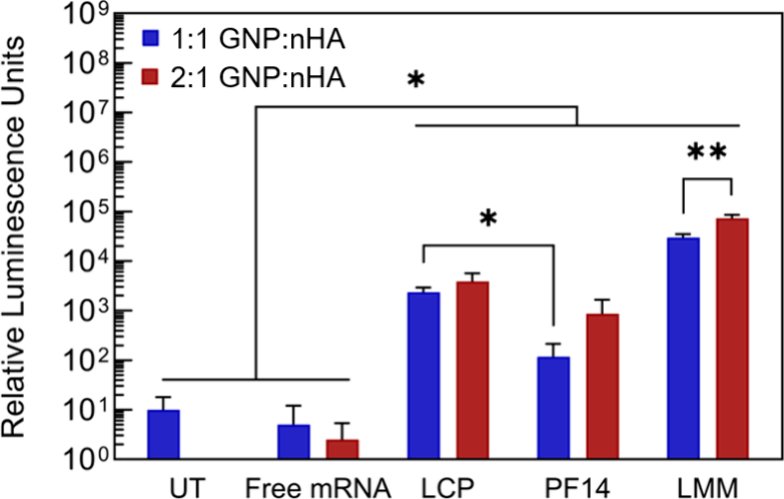
Transfection efficiency of mRNA-nanoparticles after incorporation into nanocomposites of different gelatin-to-hydroxyapatite nanoparticle ratios (GNP:nHA) tested with MC3T3 cells. UT = untreated, LCP = lipid-coated calcium phosphate, PF14 = PepFect 14, and LMM = Lipofectamine Messenger Max.

The incorporation of mRNA-NPs into a 3D nanocomposite has the potential to retain mRNA locally, but Figon, on the other hand, might also hinder transfection. Therefore, we assessed the transfection efficiency of the different mRNA-NPs (LCP, PF14, and LMM) when added to GNP-nHA nanocomposites. All mRNA-NPs led to the expression of luciferase above background, independent of the GNP-to-HA ratio of the nanocomposite (Figure 5). Among the formulations, LMM consistently yielded the highest expression for both 1:1 and 2:1 nanocomposites, while PF14 showed the lowest expression, a difference that was statistically significant for the 1:1 formulation. When comparing the expression in 1:1 vs 2:1 nanocomposites, LMM showed lower expression in the 1:1 formulation. The transfection efficiency for the LCP-containing nanocomposite exceeded the one of PF14 and was independent of the GNP-to-HA ratio. This observation is in line with the above-described results of 2D cell culture, where transfection efficiency decreased with increased nHA concentration for PF14 and LMM. These results, in combination with the previously obtained 2D culture results, stress the importance of mRNA-NP stability.

Overall, our results show that GNP-nHA nanocomposites are cytocompatible and allow for the delivery of mRNA-NPs. In the LCP-containing nanocomposites we demonstrate the use of calcium phosphate as a bifunctional material: first, as a building block of the bulk material to mechanically reinforce the nanocomposite and potentially stimulate osteogenic differentiation due to their osteoconductive properties^19^ and, second, as a transfection agent that protects mRNA and allows for its efficient delivery into cells. Notably, these two functions pose seemingly opposite requirements; i.e., as a building block the nanoparticles should strongly interact with each other to form a stable nanocomposite while mRNA delivery necessitates that nanoparticles dissociate from the nanocomposite to be available for internalization by cells. The (in)stability of the nanocomposite depends on nanoparticle properties such as size, charge, and degradability. Nanosized particles have been shown to form more stable nanocomposites compared to microsized particles.^16^ Similarly, nonspherical nanoparticles can impede the self-healing ability of nanocomposites.^12^ With regard to mRNA transfection, both size and charge have been reported to affect transfection efficiency. In general, smaller mRNA-NPs are often found to outperform larger (micro)particles which may be related to differences in internalization routes for small and large particles.^46,47^ Yet, which size leads to the most efficient transfection may also be dependent on the species as a recent study in mice and nonhuman primates suggests.^48^ Positively charged transfection agents generally enhance cellular uptake, thereby promoting transfection, but are often also more cytotoxic. Of note, the zeta potential of mRNA-NPs is usually measured in water or PBS while in biological fluids a protein corona is formed on the surface of nanoparticles, influencing their charge, structure, interaction with cells, and ultimately transfection efficiency.^49−51^ Interestingly, while both peptide-based (PF14) and LCP-based mRNA-NPs had positive zeta potentials, peptide- and lipid-based mRNA-NPs (PF14 and LMM) were more negatively affected by the presence of nHA compared to LCP-based mRNA-NPs. Moreover, we observed that metabolic activity of MC3T3 cells increased upon exposure to nHA for 24 h in 2D, as reported previously for bone-derived cells.^52^ Importantly, an increase in metabolic activity can point either to a higher cell number or to cellular stress and subsequent cell death at later time points.^43^ Although LMM showed lower transfection efficiency with higher nHA content, these LMM-complexed mRNA-NPs still led to the highest expression compared with the other mRNA-NPs. However, LMM has an unfavorable toxicity profiles for *in vivo* applications, and its application is thus restricted to in vitro use.^53^ LMM merely serves as a best-case scenario in terms of mRNA delivery and expression. By comparison, LCPs showed stable expression independent of the nHA content, which is likely due to the protective effect of the calcium phosphate core and asymmetrical lipid bilayer of LCPs. Moreover, LCPs show low cytotoxicity in vitro (Figures 4A and S3A) likely due to a favorable balance of charged, protonatable, and neutral lipids in the outer shell of the asymmetrical lipid bilayer.^31^ Since LCPs are a relatively new type of mRNA-NPs, information on their biocompatibility is still scarce, but no adverse effects were reported in previously reported *in vivo* studies.^24,30,31^ Overall, these results highlight the importance of sufficient mRNA protection and stability of mRNA-NPs for the functionalization of biomaterials.

## Conclusion

mRNA has emerged as a new class of therapeutic agents to enhance the regenerative performance of biomaterials. Here, we investigated the cytocompatibility of nanocomposites made of GNPs and nHA for the delivery of different mRNA-NPs, and we demonstrated successful mRNA transfection using these new nanocomposites. Moreover, we showed that calcium phosphate nanoparticles can be used to mechanically reinforce both nanoparticle-based nanocomposites and complex mRNA. Calcium phosphate mRNA-NPs were less sensitive to the presence of inorganic biomaterials compared to peptide- and lipid-based transfection agents, likely due to better protection against decomplexation. Importantly, our results highlight the need for sufficient mRNA protection and stability of mRNA-NPs to maintain their transfection efficiency when incorporated into an inorganic biomaterial. Alternative complexation strategies such as hybrid nanoparticles should be further explored in the future to increase the stability of mRNA-NPs upon incorporation into biomaterials.

## Supporting Information Available

Hydrodynamic size distribution of gelatin, hydroxyapatite, and mRNA-nanoparticles; structural analysis of hydroxyapatite nanoparticles; hydrogel self-healing after consecutive strain–recovery cycles; and effect of hydroxyapatite nanoparticles on the cytocompatibility and transfection efficiency of mRNA-nanoparticles in hBMSCs (PDF)
