# Supplementary material for: Local mRNA Delivery from Nanocomposites Made of Gelatin and Hydroxyapatite Nanoparticles
Source: ACS Appl Mater Interfaces. 2024 Sep 16;16(38):50497–506. doi: 10.1021/acsami.4c12721 (PMC11440464; doi:10.1021/acsami.4c12721)
Supplement: Supplementary file 1 — am4c12721_si_001.pdf [file am4c12721_si_001.pdf]

Supporting Information

Local mRNA delivery from nanocomposites made of gelatin and hydroxyapatite nanoparticles

*Lea Andrée[1 †], Rik Oude Egberink[2 †], Renée Heesakkers[1], Ceri-Anne E. Suurmond[1], Lucas S. Joziasse[1], Masoomeh Khalifeh[2], Rong Wang[1], Fang Yang[1], Roland Brock[2,3 † *], Sander C. G. Leeuwenburgh[1 † *]*

[1] Department of Dentistry—Regenerative Biomaterials, Radboud University Medical Center, Philips van Leydenlaan 25, 6525 EX Nijmegen, The Netherlands

[2] Department of Medical BioSciences, Radboud University Medical Center, Geert Grooteplein 28, 6525 GA Nijmegen, The Netherlands

[3] Department of Medical Biochemistry, College of Medicine and Medical Sciences, Arabian Gulf University, Manama 329, Bahrain

† Contributed equally

* Corresponding authors

Email: sander.leeuwenburgh@radboudumc.nl and roland.brock@radboudumc.nl

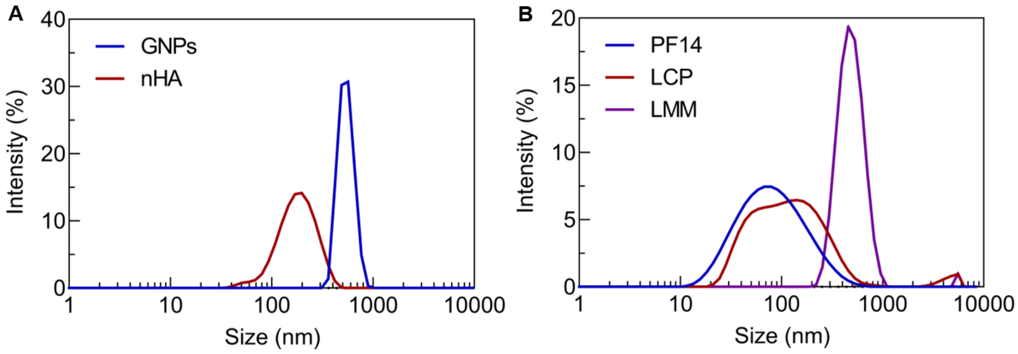

Figure S1. Hydrodynamic size distribution of (A) gelatin and hydroxyapatite nanoparticles and (B) mRNA-nanoparticles as measured by dynamic light scattering. PF14 = PepFect 14, LCP = lipid-coated calcium phosphate and LMM = Lipofectamine Messenger Max.

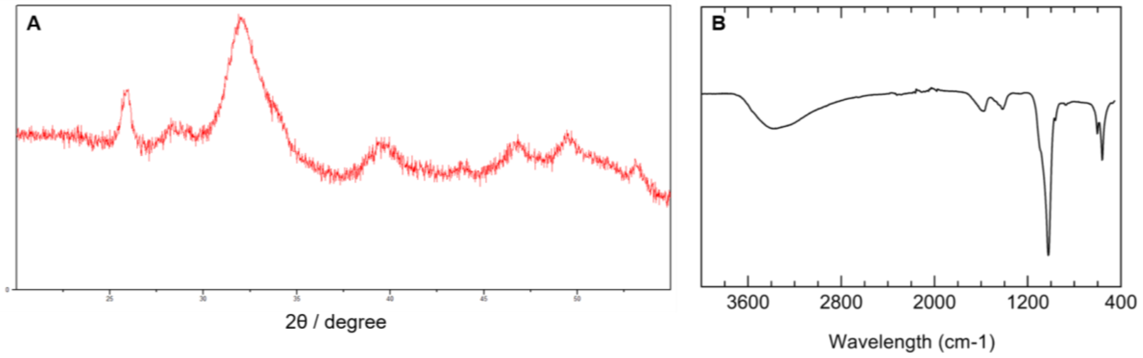

Figure S2. Structural analysis of hydroxyapatite nanoparticles by (A) X-ray diffraction and (B) Fourier-transform infrared spectroscopy.

Table S1. Hydrogel self-healing after consecutive strain-recovery cycles

GNP:nHA ratio	1st recovery (%)	2nd recovery (%)	3rd recovery (%)
1:1	60 ± 5	91 ± 1	95 ± 2
2:1	57 ± 16	93 ± 3	99 ± 5
4:1	67 ± 1	95 ± 5	98 ± 2

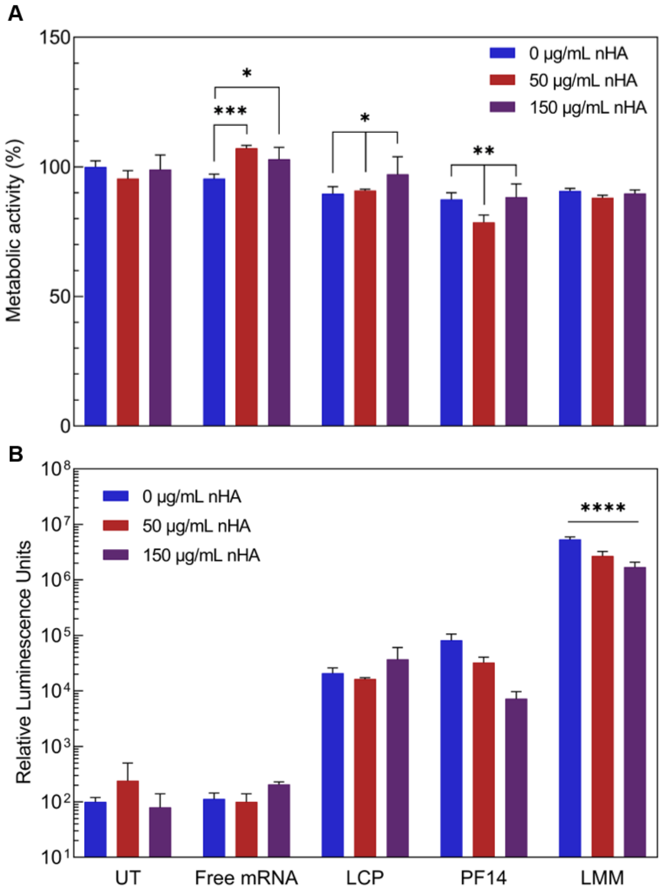

Figure S3. Effect of hydroxyapatite nanoparticles on (A) the cytocompatibility and (B) transfection efficiency of mRNA-nanoparticles in hBMSCs. UT = untreated, LCP = lipid-coated calcium phosphate, PF14 = PepFect 14 and LMM = Lipofectamine Messenger Max.
